# Body fat distribution, in particular visceral fat, is associated with cardiometabolic risk factors in obese women

**DOI:** 10.1371/journal.pone.0185403

**Published:** 2017-09-28

**Authors:** Theodora W. Elffers, Renée de Mutsert, Hildo J. Lamb, Albert de Roos, Ko Willems van Dijk, Frits R. Rosendaal, J. Wouter Jukema, Stella Trompet

**Affiliations:** 1 Department of Clinical Epidemiology, Leiden University Medical Center, Leiden, The Netherlands; 2 Department of Cardiology, Leiden University Medical Center, Leiden, The Netherlands; 3 Department of Radiology, Leiden University Medical Center, Leiden, The Netherlands; 4 Department of Human Genetics, Leiden University Medical Center, Leiden, The Netherlands; 5 Department of Medicine, Division Endocrinology, Leiden University Medical Center, Leiden, The Netherlands; 6 Department of Gerontology and Geriatrics, Leiden University Medical Center, Leiden, The Netherlands; Tongji Med College, HUST, CHINA

## Abstract

**Background:**

Body fat distribution is, next to overall obesity, an important risk factor for cardiometabolic outcomes in the general population. In particular, visceral adipose tissue (VAT) is strongly associated with cardiometabolic risk factors. Since it is unclear whether body fat distribution is also important in men and women with obesity we investigated the associations between measures of body fat distribution and cardiometabolic risk factors in men and women with obesity.

**Methods:**

In this cross-sectional analysis of obese men and women (BMI≥30 kg/m^2^) included in the Netherlands Epidemiology of Obesity Study, waist:hip ratio(WHR), waist circumference, and MRI-based abdominal subcutaneous adipose tissue (aSAT) and VAT were determined. Associations between measures of body fat distribution and presence of ≥1 risk factor, such as hypertension or hypertriglyceridemia, were examined using logistic regression analyses; stratified by sex and adjusted for age, ethnicity, education, tobacco smoking, alcohol consumption, physical activity and depending on the association additionally for total body fat or VAT.

**Results:**

We included 2,983 obese individuals (57% women) with a mean age of 56 and standard deviation (SD) of 6 and mean BMI of 34.0 kg/m^2^ (4.0), after exclusion of individuals with missing values of cardiometabolic risk factors (n = 33). 241 individuals were obese without other cardiometabolic risk factors. In obese women, all measures of body fat distribution except aSAT (OR per SD:0.76, 95%CI: 0.53, 1.10) were associated with having ≥1 cardiometabolic risk factor, of which VAT most strongly associated (5.77; 3.02, 11.01). In obese men, associations of body fat distribution and the presence of cardiometabolic risk factors were attenuated. (e.g. VAT:1.42; 0.84, 2.41).

**Conclusions:**

In obese women, but less so in men, measures of body fat distribution, of which VAT most strongly, are associated with cardiometabolic risk factors.

## Introduction

Obesity has become a major health problem and in several countries its prevalence keeps rising.[1, 2] It has been estimated that obesity was responsible for 3.4 million deaths in 2010 worldwide.[3] Obesity is an important risk factor for the development of cardiovascular diseases (CVD) through cardiometabolic abnormalities such as insulin resistance and hypertension. In addition to overall obesity, body fat distribution, abdominal obesity in particular, has emerged as an important risk factor for type 2 diabetes and CVD in the general population.[4, 5] It has been proposed that the excess risk of cardiometabolic disease associated with abdominal obesity is due to the presence of large amounts of visceral adipose tissue (VAT), which is highly metabolically active.[6] Next to insulin resistance, VAT has also been associated with hypertension and subclinical atherosclerosis in the general population.[7] Since body fat distribution is sexually dimorphic and sex hormones may play a role in the adverse effects of VAT, associations of VAT with cardiometabolic risk factors may differ between men and women.[8, 9]

It is unclear whether these associations of abdominal adiposity with cardiometabolic risk factors are also present in obese individuals. Although obesity is associated with adverse cardiometabolic effects and CVD, not all obese individuals have cardiometabolic abnormalities. It has been suggested that 10–30% of obese individuals do not have obesity-associated cardiometabolic disorders.[10–14] This ‘healthy obese’ phenotype has been associated with a lower amount of visceral and ectopic fat relative to subcutaneous fat.[15–17] It thus appears that abdominal adiposity, and especially amount of VAT is also important in individuals.[18, 19] Therefore, we aimed to investigate the associations between measures of abdominal adiposity with several cardiometabolic risk factors in obese men and women in the NEO Study.

## Methods

### Study design and population

The Netherlands Epidemiology of Obesity (NEO) study is a population-based, prospective cohort study designed to investigate pathways that lead to obesity-related diseases, including 6 671 individuals. Men and women aged between 45 and 65 years with a self-reported BMI of 27 kg/m2 or higher living in the area of greater Leiden (in the West of The Netherlands) were eligible to participate in the NEO study. In addition, all inhabitants aged between 45 and 65 years from one municipality (Leiderdorp) were invited irrespective of their BMI, allowing for a reference distribution of BMI. Individuals were invited to a baseline visit at NEO study centre of the LUMC after an overnight fast. At the time of inclusion, individuals completed a screening form, asking about anything that might create a health risk or that might interfere with imaging (most notably metallic devices, or claustrophobia). An additional contraindication for magnetic resonance imaging (MRI) was a body circumference of more than 1.70 m. Of the individuals without contra-indications for MRI, approximately 35% were randomly selected to undergo MRI. Prior to the study visit, individuals completed a questionnaire at home with demographic, lifestyle, and clinical information. At the study centre all individuals underwent an extensive physical examination, including anthropometry and blood sampling. In the present study, individuals without obesity and individuals with missing values of waist circumference, glucose, triglycerides, HDL-cholesterol concentrations, or blood pressure were excluded. Further details of the study design and population have been described in detail elsewhere.[20] The Medical Ethical Committee of the Leiden University Medical Center (LUMC) approved the design of the study and all individuals gave their written informed consent.

### Data collection

The ethnicity of individuals was self-identified in eight categories on the questionnaire and then grouped into white and other. Level of education was reported in 10 categories according to the Dutch education system and grouped as low (none, primary school of lower vocational education) versus high education. Tobacco smoking was categorized into current smoker, former smoker, or never smoker. Alcohol consumption was reported using a food frequency questionnaire and calculated into grams/day.[21] Physical activity was reported by the individuals using the Short Questionnaire to Assess Health-enhancing physical activity (SQUASH).[22] We calculated the energy expended during physical activity in leisure time in hours per week of metabolic equivalents (MET-h/week). Individuals were asked to bring all the medication they were currently using to the study visit and to report their medical history of diabetes or CVD. Brachial blood pressure was measured in a seated position on the right arm using a validated automatic oscillometric device (OMRON, Model M10-IT, Omron Health Care Inc, IL, USA). Blood pressure was measured three times with 5 minutes rest between consecutive measurements. The mean systolic and diastolic blood pressure were calculated. Blood plasma was sampled after an overnight fast of 10 hours. Fasting glucose, triglyceride and high-density lipoprotein concentrations were measured with standard methods in the central clinical chemistry laboratory of the LUMC.[20]

### Measures of body fat

Height and weight were measured without shoes and 1 kg was subtracted from the weight to correct for clothing. BMI was calculated by dividing the weight in kilograms by the height in meters squared. Obesity is defined as BMI ≥ 30 kg/m^2^. The waist circumference was measured with a horizontally placed flexible tape in the middle of the distance between the lowest rib and the iliac crest. The hip circumference (HC) was measured at the maximum circumference of the buttocks. The waist-hip-ratio (WHR) was calculated by dividing the waist circumference by the HC. With a bio-impedance device (TBF-310, Tanita International Division, UK) total body fat (TBF) was estimated. Abdominal subcutaneous adipose tissue (aSAT) and VAT were assessed by MR imaging (1.5 Tesla MR imaging, Philips Medical Systems) using a turbo spin echo imaging protocol in a subgroup of 2580 individuals. Three transverse images with a slice thickness of 10 mm were obtained during a breath-hold at the level of the fifth lumbar vertebra. The fat depots were converted from the number of pixels to centimeters squared. In the analyses, the average of the three slices was used.

### Cardiometabolic risk factors

To define different cardiometabolic risk factors, we used four components of the definition of metabolic syndrome as proposed by the National Cholesterol Education Program (NCEP) Adult Treatment Panel III (ATPIII), with minor modifications as stated in the American Heart Association (AHA) and the National Heart, Lung, and Blood Institute (NHLBI) statement.[23] We used 1) raised serum triglyceride concentrations (≥1.7 mmol/L) or on drug treatment to reduce triglyceride concentrations; 2) reduced serum HDL-cholesterol concentrations (<1.03 mmol/L for men, <1.3 mmol/L for women) or on drug treatment to elevate HDL-cholesterol; 3) raised blood pressure (≥130 mmHg systolic/≥85 mmHg diastolic) or on antihypertensive drug treatment; 4) raised fasting plasma glucose concentrations (≥5.56 mmol/L) or on drug treatment to lower glucose concentrations.

### Statistical analysis

Baseline characteristics are presented as mean (SD), median (interquartile range) or as percentage. We standardized the measures of body fat to a mean of zero with a standard deviation of one. Then we performed logistic regression analyses and calculated odds ratios (ORs) and 95% confidence intervals, per standard deviation of measure of body fat, on having at least one risk factor compared with men and women without any other cardiometabolic risk factors than obesity.

Crude associations were adjusted for sex, age, ethnicity, smoking, alcohol intake, education level, physical activity and statin use. Associations of WHR, waist circumference and VAT were additionally adjusted for total body fat and associations of aSAT were additionally adjusted for VAT. Data were analysed using STATA (Statacorp, College Station, Texas, USA), version 14.

## Results

After excluding individuals with missing data on fasting plasma glucose concentrations or glucose lowering therapy (n = 26), blood pressure or use of antihypertensive therapy (n = 5) and serum triglyceride concentrations or use of medication to reduce triglyceride concentrations (n = 2), we ultimately included 2,983 individuals in our analyses of which in 1,071 individuals VAT and aSAT measurements were available. The baseline characteristics are shown in Table 1. Of our study population, 241 individuals did not have any cardiometabolic risk factors and 2,742 individuals had at least one cardiometabolic risk factor. However, 3% of individuals in the group without cardiometabolic risk factors did use lipid lowering medication. Next to 8% participants without any cardiometabolic risk factors, 26% had one risk factor, 34% had two risk factors, 21% had three risk factors and 11% had 4 risk factors. Compared with individuals without any risk factors, individuals with at least one other risk factor were older, more often men, more often former or current smoker and had a higher alcohol intake. There was no difference in physical activity between the groups.

**Table 1:** Baseline characteristics of the study population.

	Cardiometabolic health status	
	0 risk factors (n = 241)	≥1 risk factor (n = 2,742)
Age, years	53 (6)	56 (6)
Sex, men, %	23	45
Ethnicity, white, (%)	95	94
Smoking		
Never, %	41	32
Former, %	45	53
Current, %	14	16
Alcohol intake, g/day	4.3 (1.0–14.5)	7.8 (1.0–21.7)
Physical activity (MET-hour/week)	24.8 (15.0–42.5)	24.5 (11.1–44.0)
Education level, low, (%)^a^	27	33
History of CVD, %	3	9
BMI, kg/m^2^		
Men	32.7 (2.9)	33.3 (3.3)
Women	33.5 (3.3)	34.7 (4.5)
Waist:hip ratio		
Men	0.99 (0.1)	1.02 (0.1)
Women	0.88 (0.1)	0.90 (0.1)
Waist circumference, cm		
Men	112.9 (8.3)	115.2 (9.3)
Women	104.0 (9.1)	107.4 (11.0)
aSAT, cm^2^		
Men	360.1 (110.6)	330.3 (87.1)
Women	448.0 (95.4)	432.9 (93.3)
VAT, cm^2^		
Men	148.5 (69.6)	180.4 (64.8)
Women	90.0 (30.7)	132.4 (51.2)
Systolic blood pressure, mmHg	116.3 (7.8)	135.7 (16.8)
Diastolic blood pressure, mmHg	76.6 (5.5)	87.2 (10.1)
Use of antihypertensive therapy, *%*	0	45
Triglycerides, mg/Dl	88.6 (67.3–111.6)	130.2 (93.0–176.3)
HDL-cholesterol, mg/dL		
Men	51.1 (7.9)	44.7 (10.9)
Women	65.5 (11.4)	56.4 (14.2)
Use of lipid lowering therapy, %	3	21
Fasting glucose, mg/dL	92.8 (5.5)	109.1 (24.0)
Use of glucose lowering therapy, *%*	0	10

Data are presented as mean (SD), median (25^th^, 75^th^ percentiles), or percentages. MET, metabolic equivalent of task during leisure time; BMI, body mass index; VAT, visceral adipose tissue; aSAT, abdominal subcutaneous adipose tissue. CVD, cardiovascular disease; HDL, high-density lipoprotein.

^a^ lower education: none, primary school, lower vocational education

**Fig 1:**
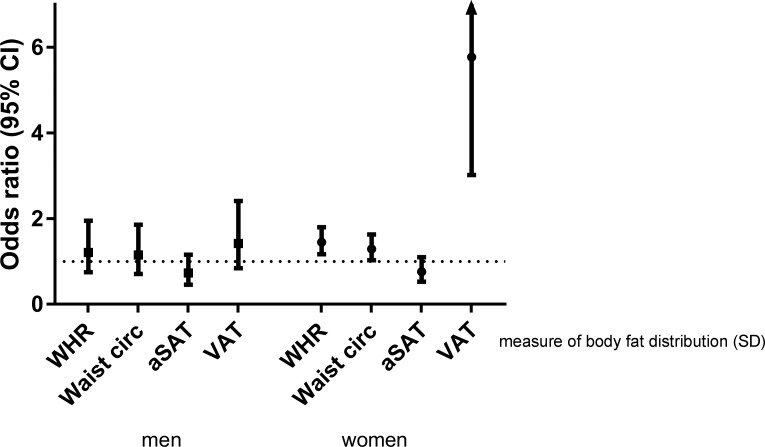
Association of measures of body fat distribution on having at least one cardiometabolic risk factor. Data are presented as odds ratio (95% CI) per standard deviation of measure of body fat distribution in men and women. WHR, waist:hip ratio; WC, waist circumference; aSAT, abdominal subcutaneous adipose tissue; VAT, visceral adipose tissue adjusted for age, ethnicity, education, tobacco smoking, alcohol consumption and physical activity. Associations of WHR, WC and VAT are additionally adjusted for total body fat and associations of aSAT additionally for VAT.

Table 2 and Fig 1 show ORs and 95% confidence intervals per standard deviation of measure of body fat distribution on having at least one cardiometabolic risk factor in the whole study population and in men and women separately. In the whole study population, one SD higher WHR (0.1) was associated with an OR of 1.40 on having at least one cardiometabolic risk factor compared with individuals without any risk factors (95%CI: 1.15, 1.70). One SD higher waist circumference (11cm) was associated with an OR of 1.29 (1.05, 1.59) and one SD higher VAT (64cm^2^) most strongly with an OR of 2.91 (1.94, 4.36) on having at least one cardiometabolic risk factor. In the whole study population, no association was found between aSAT and having at least one cardiometabolic risk factor (OR: 0.79; 95%CI: 0.60, 1.04). Also, there was a clear dose-response between number of cardiometabolic risk factors and measures of body fat distribution, with higher WHR, waist circumference and VAT associated with higher number of risk factors. This was not visible for aSAT. (Data not shown)

**Table 2:** Odds ratios per SD of measures of body fat distribution on having at least one cardiometabolic risk factor.

	All		Men		Women		
Fat measure (SD)	Crude	Adjusted^1^	Crude	Adjusted^1^	Crude	Adjusted^1^	p-value interaction^2^
	n = 2,981		n = 1,284		n = 1,697		
WHR (0.1)	1.79 (1.56, 2.06)	1.40 (1.15, 1.70)	1.76 (1.16, 2.66)	1.21 (0.75, 1.95)	1.58 (1.28, 1.95)	1.45 (1.17, 1.80)	0.380
WC (11 cm)	1.66 (1.43, 1.92)	1.29 (1.05, 1.59)	1.39 (0.96, 2.00)	1.15 (0.71, 1.86)	1.43 (1.20, 1.70)	1.29 (1.03, 1.63)	0.397
	n = 1,071		n = 536		n = 535		
aSAT (105 cm^2^)	0.69 (0.56, 0.85)	0.79 (0.60, 1.04)	0.69 (0.45, 1.07)	0.73 (0.46, 1.16)	0.84 (0.63, 1.12)	0.76 (0.53, 1.10)	0.565
VAT (64 cm^2^)	3.25 (2.30, 4.58)	2.91 (1.94, 4.36)	1.81 (1.12, 2.94)	1.42 (0.84, 2.41)	5.00 (2.91, 8.60)	5.77 (3.02, 11.01)	0.002

Data are presented as odds ratio (95% CI) per standard deviation of measure of body fat distribution; WHR, waist:hip ratio; WC, waist circumference; aSAT, abdominal subcutaneous adipose tissue; VAT, visceral adipose tissue.

1: adjusted for age, sex (in all), ethnicity, education, tobacco smoking, alcohol consumption and physical activity. Associations of WHR, WC and VAT are additionally adjusted for total body fat and associations of aSAT additionally for VAT

2: interaction was tested for the adjusted model

In women, 1 SD higher WHR was associated with an OR of 1.45 on having at least one cardiometabolic risk factor compared with individuals without any risk factors (95% CI: 1.17, 1.80), 1 SD higher waist circumference with an OR of 1.29 (1.03, 1.63) and 1 SD higher VAT with an OR of 5.77 (3.02, 11.01). Abdominal SAT was not associated with an OR of 0.76 (0.53, 1.10) per SD on having at least one cardiometabolic risk factor.

In men, the associations of the measures of body fat distribution and cardiometabolic risk factors were much weaker than in women or even absent. In women one SD higher VAT (64.0 cm^2^) was associated with an OR of 5.77 (95% CI: 3.02, 11.01) on having at least one cardiometabolic risk factor, while in men one SD higher VAT was associated with an OR of 1.42 (0.84, 2.41) on having at least one cardiometabolic risk factor. (p-value interaction: 0.002) In men, WHR (OR: 1.21; 95%CI: 0.75, 1.95), waist circumference (1.15; 0.71, 1.86) and aSAT (0.73; 0.46, 1.16) were not associated with an increased cardiometabolic risk.

## Discussion

In this cross-sectional study, we examined several measures of body fat distribution in relation to cardiometabolic risk factors in obese men and women participating in the NEO study. In obese women, WHR, waist circumference and VAT were associated with an increased cardiometabolic risk, whereas aSAT was not. Furthermore, in obese women, VAT was most strongly associated with an increased cardiometabolic risk. In obese men, associations between measures of body fat distribution and cardiometabolic health were much weaker, if present at all. Several studies in the general population have also shown associations of abdominal adiposity, and visceral adiposity in particular, with cardiometabolic risk factors (reviewed in[6]) In the Framingham Heart Study, it was found that also in obese individuals, VAT was associated with hypertension, impaired fasting plasma glucose and the metabolic syndrome.[24] In a study in obese adults, VAT was associated with impaired fasting plasma glucose and type 2 diabetes mellitus, whereas general adiposity was not.[25]

VAT could be associated with increased cardiometabolic risk through several mechanisms. VAT is characterised by a high rate of lipolysis, resulting in an excess production of free fatty acids. These free fatty acids are released into the portal circulation and transported to the liver, which could result in excess intra-hepatic fat, a risk factor for cardiometabolic disease.[26, 27] In addition, VAT has a high secretion rate of growth factors, cytokines, and hormones that are involved in the pathogenesis of cardiometabolic diseases.[28–30] Furthermore, when adipocytes become larger with an increase in the amount of VAT, they also become more dysfunctional, for example through hypoxia, which leads to the increased release of free fatty acids and cytokines.[31]

We observed clear differences in associations of fat distribution and cardiometabolic risk factors between men and women. In obese women, measures of body fat distribution were associated with cardiometabolic health status, while in men they were not or only weakly associated. Differences in associations of measures of body fat distribution and cardiometabolic risk factors between men and women have previously been reported from other studies. In the Framingham Heart Study, in both obese men and women, VAT was associated with hypertension and the metabolic syndrome. The association of VAT with impaired fasting plasma glucose was only present in obese women.[24] A study with older individuals observed that VAT was stronger associated with diabetes in women than in men. However, this study did not investigate obese individuals in particular.[32] Another study of normal-weight individuals observed that VAT was associated with cardiovascular risk factors only in women, but not in men.[33] The exact explanation for these sex differences is to our knowledge not known, but sex steroids likely play a role.[8] Body fat distribution is sexually dimorphic and it is well-known that men accrue more visceral fat and women accrue more subcutaneous fat in general.[9] After the menopause, adipose tissue of women shifts toward the visceral fat depot, likely due to decreasing oestrogen levels.[34] A study with 68 individuals showed that increasing VAT was associated with an increased contribution of VAT lipolysis to hepatic free fatty acid delivery and that this association was stronger in women than in men.[35] Also, some pituitary hormones are known to influence adipocyte function. Prolactin and growth hormone have both been shown to stimulate lipolysis and the effects of growth hormone seem to differ between internal or subcutaneous adipose tissue sites.[36] Furthermore, it is known that these pituitary hormones can be influenced by sex hormones or have different mRNA levels in men and women.[37, 38] Because of their influence on both the distribution and the function of adipose tissue, it is thus likely that sex hormones play an important role in the observed differences between men and women.[39, 40]

A strength of our study is the large study population (n = 2,983) and the extensive phenotyping of the individuals at baseline. Despite the extensive measurements of potential confounding factors in the NEO study, we cannot exclude the possibility of residual confounding. Furthermore, in our study VAT and aSAT were directly assessed by MR imaging. A weakness of this study is that we cannot determine causal relations, because of the observational cross-sectional design. Furthermore, our study population consists mostly of white individuals, and associations between fat depots and cardiometabolic risk factors might differ between ethnic groups. Also, VAT was measured using three transverse slices at the level of the fifth vertebra and then converted to centimetres squared, which does not completely correspond with total VAT volume.[41]

In conclusion, our results are in line with previous literature, indicating that abdominal adiposity is an important determinant of cardiometabolic health. On top of previous literature, we showed that in obese women, but less so in obese men, VAT is most strongly associated with cardiometabolic risk factors, compared with the other measures of body fat distribution. Future studies should aim at unravelling the underlying mechanisms of the detrimental metabolic effects of visceral fat in women.
